# Breast cancer histopathological images classification based on deep semantic features and gray level co-occurrence matrix

**DOI:** 10.1371/journal.pone.0267955

**Published:** 2022-05-05

**Authors:** Yan Hao, Li Zhang, Shichang Qiao, Yanping Bai, Rong Cheng, Hongxin Xue, Yuchao Hou, Wendong Zhang, Guojun Zhang

**Affiliations:** 1 Department of Mathematics, Taiyuan Normal University, Taiyuan, China; 2 School of Information and Communication Engineering, North University of China, Taiyuan, China; 3 Department of Mathematics, School of Science, North University of China, Taiyuan, China; 4 Data Science and Technology, North University of China, Taiyuan, China; 5 School of Instrument and Electronics, Key Laboratory of Dynamic Testing Technology, North University of China, Taiyuan, China; University of South Carolina, UNITED STATES

## Abstract

Breast cancer is regarded as the leading killer of women today. The early diagnosis and treatment of breast cancer is the key to improving the survival rate of patients. A method of breast cancer histopathological images recognition based on deep semantic features and gray level co-occurrence matrix (GLCM) features is proposed in this paper. Taking the pre-trained DenseNet201 as the basic model, part of the convolutional layer features of the last dense block are extracted as the deep semantic features, which are then fused with the three-channel GLCM features, and the support vector machine (SVM) is used for classification. For the BreaKHis dataset, we explore the classification problems of magnification specific binary (MSB) classification and magnification independent binary (MIB) classification, and compared the performance with the seven baseline models of AlexNet, VGG16, ResNet50, GoogLeNet, DenseNet201, SqueezeNet and Inception-ResNet-V2. The experimental results show that the method proposed in this paper performs better than the pre-trained baseline models in MSB and MIB classification problems. The highest image-level recognition accuracy of 40×, 100×, 200×, 400× is 96.75%, 95.21%, 96.57%, and 93.15%, respectively. And the highest patient-level recognition accuracy of the four magnifications is 96.33%, 95.26%, 96.09%, and 92.99%, respectively. The image-level and patient-level recognition accuracy for MIB classification is 95.56% and 95.54%, respectively. In addition, the recognition accuracy of the method in this paper is comparable to some state-of-the-art methods.

## Introduction

Cancer has always been a serious threat to human life and health [1]. In 2020, there were 19.29 million new cancer cases worldwide, including 4.57 million new cancer cases in China (2.48 million males and 2.09 million females), accounting for 23.7% of the global total. One of the most obvious changes in the latest cancer data in the world in 2020 is the rapid growth of new cases of breast cancer. It has replaced lung cancer as the world’s largest cancer (2.26 million cases of breast cancer and 2.2 million cases of lung cancer) [2]. Early diagnosis and effective treatment of breast cancer is the key to improving the survival rate of patients.

Compared with X-rays, mammography, magnetic resonance and other diagnostic methods, histopathological images can provide more important basis for cancer diagnosis and are considered as the gold standard for breast cancer diagnosis. The Whole Slide Image (WSI) of histopathological images usually with a large size, ranging from 40000 to 100000 pixels. Manual diagnosis of histopathological images is time-consuming, labor-intensive and error prone, which depends on the degree of concentration and fatigue of pathologists, it also requires a lot of prior knowledge and diagnostic experience. Misdiagnosis of breast cancer can cause very serious consequences, especially when a patient with malignant tumor is diagnosed as a benign, which may lead to missing the best time for diagnosis and treatment, and even makes the patients lose their lives. At present, the number of histopathological images produced every day is numerous, and the number of experienced pathologists is far from enough, which has seriously hindered the early diagnosis of breast cancer. In order to solve these problems, researches on Computer Aided Diagnosis (CAD) emerge in endlessly. CAD can not only improve the efficiency of diagnosis, but also reduce the workload of pathologists while providing more objective diagnosis results.

Some researchers employ handcrafted features for breast cancer histopathological images recognition. Spanhol et al. [3] proposed a publicly available breast cancer histopathology dataset called BreaKHis, six kinds of features including GLCM were used for the classification of this dataset, and the accuracy range from 80% to 85%. Belsare et al. [4] extracted features such as GLCM, graph run length matrix and Euler number for breast cancer histopathological images recognition. In our previous work [5], we explored the application of 9 feature descriptors such as GLCM in breast cancer histopathological image recognition. Anuranjeeta et al. [6] proposed a breast cancer recognition method based on morphological features. 16 morphological features were extracted, and 8 classifiers were used and the accuracy is about 80%. Sharma et al. [7] first segmented the nuclei region of the images and the parameter-free threshold adjacency statistics (PFTAS) features were extracted, then random forest (RF) was used for benign and malignant classification of breast cancer histopathological images. Carvalho et al. [8] used phylogenetic diversity indexes to characterize the types of breast cancer. Boumaraf et al. [9] fused Zernike moments features, Haralick features, and color histogram features for binary and eight classes classification of breast cancer histopathological images.

In recent years, the excellent performance of deep learning in images recognition has aroused the interest of a large number of researchers, especially CNNs. The evolution of these models prompted researchers to develop CNNs based CAD models and apply them to cancer diagnosis, such as breast cancer, lung cancer, prostate cancer, cervical cancer and liver cancer. In a broad sense, there are two kinds of CNNs, namely, the CNNs trained from scratch [10–15] and the pre-trained CNNs [16–21]. Spanhol et al. [10] trained a CNN with different image patches generation strategies from scratch based on a variant of AlexNet [22], and combined these patches for final classification of the BreaKHis dataset. Considering the cost of computation, the problem of convergence and the shortage of high quality labeled histopathological images, it is not the most practical strategy to train the model from scratch. In addition, the training of the model may be very time-consuming for a large number of data due to hardware constraints. To save training time, the authors of [10] also used pre-trained CNN to extract Deep Convolutional Activation Features (DeCAF), and then learned classifiers for new classification tasks [16]. The experimental results proved that the recognition accuracy of deep learning is significantly higher than their previous work [3]. Compared with the CNN trained from scratch, transfer learning from pre-trained models provides better initialization weights than random initialization, which speeds up the training of the models. Moreover, transfer learning makes it possible to build deep networks based on a small amount of data.

Many researchers also use CNN as a feature extractor to classify the extracted deep features using different machine learning methods. For example, Li et al. [23] used ResNet50 [24] to extract the features of image patches with different sizes, 3-norm was used for feature fusion, and finally used SVM for classification. Kausar et al. [25] extracted deep features by VGG16 [26] based on images obtained from Haar wavelet decomposition, and combined different features of the middle layers for breast cancer recognition. Saxena et al. [27] employed the pre-trained ResNet50 and the kernelized weighted extreme learning machine to analyze the histopathological images and solved the class imbalance problem. Man et al. [28] proposed an unsupervised anomaly detection with generative adversarial networks (AnoGAN) to screen mislabeled patches and used pre-trained DenseNet121 [29] to extract multi-layered features of the discriminative patches for breast cancer classification. Saini et al. [30] first used a deep convolution generative adversarial network for data augmentation of benign samples, and then extracted the features of different pooling layers with a variant of pre-trained VGG16, and SVM was used for classification. Li et al. [31] proposed an interleaved DenseNet with SENet (IDSNet), which used the output of the three transition layers and the fourth dense block of DenseNet121 as the input of SENet. Combined with the classification sub-network, the extracted features were cascaded for the breast cancer benign and malignant classification. Wang et al. [32] proposed a parallel dual channels model which can extract convolution features (semantic information) and capsule features (spatial information) simultaneously, the fused features were used for breast cancer recognition. The highest accuracy achieved on BreaKHis dataset at 100× was 94.52%. Murtaza et al. [33, 34] used the pre-trained AlexNet as the baseline model to extract deep features and analyzed the classification performance of six machine learning methods. Shallu and Rajesh [35] compared and analyzed the deep features extracted by pre-trained VGG16, VGG19, and ResNet50, as well as the color histogram, Hu invariant moments, and GLCM on the classification performance of breast cancer histopathological images. Experimental results show that using the pre-trained network as a feature extractor outperforms the baseline models and handcrafted features.

Part of the works explored the breast cancer histopathological images classification task with a magnification independent manner. Based on the BreaKHis dataset, Benhammou et al. [36] made a comprehensive review from four aspects: MSB, MIB, magnification specific multi-category (MSM) and magnification independent multi-category (MIM) classifications. Sharma et al. [37, 38] trained a simple 6-layer CNN model without considering magnifications based on BreaKHis, while performed independently in the test phase to determine the ability of the model in classifying the data based on the magnifications. Yari et al. [39] constructed 6 deep models with different parameter settings based on ResNet50 and DenseNet161, the highest accuracy of the MIB classification was 99.26%. Liu et al. [40] introduced Bilinear Convolutional Neural Networks (BCNNs) and compared with several other deep learning methods. The accuracy of the MIB classification is 99.24%. Boumaraf et al. [41] proposed a pre-trained ResNet18 with global contrast normalization method to automated classify breast cancer histopathological images, including MSB, MSM, MIB and MIM classifications.

However, there are still several deficiencies. First of all, the dataset was divided into the training set and the test set according to the images while not the patients in some works. It did not consider that the images of the same patient cannot be used for training and testing at the same time, so as to obtain higher recognition accuracy. Secondly, in the research of using deep learning models as feature extractors, some of them only consider the features of the fully connected layers or pooling layers, and rarely consider the features of the convolutional layers, which leads to the loss of spatial information. In addition, the data augmentation had been applied in many works, which will increase the amount and time of calculation. Considering the above problems, in this paper, the dataset was randomly divided into 70%/30% (56 patients/26 patients) under the condition that patients used to build the training set were not used for the test set. Pre-trained DenseNet201 was used to extract the convolutional layer features for breast cancer histopathological images recognition without data augmentation. Our contributions are as follows:

1. A breast cancer histopathological images recognition model with fusion of deep semantic features and GLCM features is designed, which fully utilizes the complementarity of semantic features and texture features.
2. The three-channel (R, G, B) features for GLCM is considered, which are more discriminative than grayscale features.
3. The features of deep convolution layers are extracted as deep semantic features, which retain more spatial and structural information of the images.
4. By using the pre-trained models, the demand of the model for labeled samples is reduced, which avoids the complex process of image labeling.

## Proposed method

DenseNet was proposed by Huang et al. [29]. They broke away from the stereotyped thinking of deepening the network layers (ResNet) and widening the network width (Inception) to improve the network performance, and constructed a new network structure from the perspective of features. The network structure is not complex, but very effective. DenseNet is a convolutional neural network with dense connections. In this network, the input of each layer is the union of the outputs of all previous layers, and the feature map learned by this layer will be directly transmitted to all subsequent layers as input. When CNN model is used as a feature extractor, texture features of images often come from shallow layers, including corner, edge, etc., which describe local changes of images. With the deepening of the network, the extracted features become more and more abstract, which are called semantic features and describe the global structure of images. However, the existing methods mostly emphasize the features of the fully connected layers or the pooling layers, and rarely consider the features of the convolutional layers. The nuclei of breast tumors are larger than that of normal tissues, and the nuclei are densely distributed. Compared with the features of the pooling layers and the fully connected layers, the features of the convolutional layers contain more spatial information and provide more information about the distribution of nuclei, which is of great significance for the accurate detection of breast cancer.

GLCM is a common method to describe the texture of images by studying its spatial correlation characteristics. In 1973, Haralick et al. [42] proposed using GLCM to describe texture features. The excellent ability of GLCM in breast cancer histopathological images recognition, especially for the three-channel features of the images have been discovered in [5]. In this paper, three-channel features are considered. We calculate GLCM at 0, $\frac{\pi }{4}$, $\frac{\pi }{2}$, $\frac{3\pi }{4}$ four directions with gray level of 256 and step of 1. Then, according to the GLCM, 22 features [42–44] were calculated, including autocorrelation, contrast, correlation in two forms, cluster prominence, cluster shade, dissimilarity, energy, entropy, homogeneity in two forms, maximum probability, sum of squares, sum average, sum variance, sum entropy, difference variance, difference entropy, normalized inverse difference, normalized inverse difference moment and information measures of correlation in two forms.

Given the GLCM of an image, *p*(*i*,*j*) is the (*i*,*j*)th entry in a normalized GLCM. *p*_*x*_(*i*) is the *i*th entry in the marginal-probability matrix obtained by summing the rows of *p*(*i*,*j*). *N*_*g*_ is the number of distinct gray levels in the quantized image. *μ* is the mean value of the normalized GLCM. The mean value and standard deviation for the rows and columns of the matrix are $\mu_{x}=\sum_{i}\sum_{j}i\cdot p(i,j)$, $\mu_{y}=\sum_{i}\sum_{j}j\cdot p(i,j)$, $\sigma_{x}=\sum_{i}\sum_{j}{\left(i-\mu_{x}\right)}^{2}\cdot p(i,j)$ and $\sigma_{y}=\sum_{i}\sum_{j}{\left(j-\mu_{y}\right)}^{2}\cdot p(i,j)$, respectively. The marginal-probability distribution represents as $p_{x}(i)=\sum_{j=1}^{N_{g}}p(i,j)$, $p_{y}(j)=\sum_{i=1}^{N_{g}}p(i,j)$, $p_{x+y}(k)=\sum_{i=1}^{N_{g}}\sum_{j=1}^{N_{g}}p(i,j),\;i+j=k,\;k=2,3,\cdots ,2N_{g}$, $p_{x-y}(k)=\sum_{i=1}^{N_{g}}\sum_{j=1}^{N_{g}}p(i,j)$, |*i*−*j*| = *k*, *k* = 0,1,⋯,*N*_*g*_−1. The equations of the 22 features are as follows:

$$\mathrm{Autocorrelation}\;Autoc=\sum_{i}\sum_{j}\left(ij\right)p(i,j),$$ (1)

$$\mathrm{contrast}\;Contr=\sum_{n=0}^{N_{g}-1}n^{2}(\sum_{i=1}^{N_{g}}\sum_{j=1}^{N_{g}}p(i,j)),\;|i-j|=n,$$ (2)

$$\mathrm{correlation}\;Corrp=\frac{\sum_{i}\sum_{j}(ij)p(i,j)-\mu_{x}\mu_{y}}{\sigma_{x}\sigma_{y}},$$ (3)

$$Corrm=\frac{\sum_{i}\sum_{j}\left(i-\mu_{x})(j-\mu_{y})p(i,j\right)}{\sigma_{x}\sigma_{y}},$$ (4)

$$\mathrm{cluster}\;\mathrm{prominence}\;Cprom={\sum_{i}\sum_{j}\left(i+j-\mu_{x}-\mu_{y}\right)}^{4}p(i,j),$$ (5)

$$\mathrm{cluster}\;\mathrm{shade}\;Cshad={\sum_{i}\sum_{j}\left(i+j-\mu_{x}-\mu_{y}\right)}^{3}p(i,j),$$ (6)

$$\mathrm{dissimilarity}\;Dissi=\sum_{i}\sum_{j}\left|i-j\right|\cdot p(i,j),$$ (7)

$$\mathrm{energy}\;Energ={\sum_{i}\sum_{j}p(i,j)}^{2},$$ (8)

$$\mathrm{entropy}\;Entro=-\sum_{i}\sum_{j}p(i,j)\log(p(i,j)),$$ (9)

$$\mathrm{homogeneity}\;Homop=\sum_{i}\sum_{j}\frac{1}{1+{\left(i-j\right)}^{2}}p(i,j),$$ (10)

$$Homom=\sum_{i}\sum_{j}\frac{1}{1+|i-j|}p(i,j),$$ (11)

$$\mathrm{maximum}\;\mathrm{probability}\;Maxpr=\underset{i,j}{\max}p(i,j),$$ (12)

$$\mathrm{sum}\;\mathrm{of}\;\mathrm{squares}\;Sosvh={\sum_{i}\sum_{j}\left(i-\mu \right)}^{2}p(i,j),$$ (13)

$$\mathrm{sum}\;\mathrm{average}\;Savgh=\sum_{i=2}^{2N_{g}}ip_{x+y}(i),$$ (14)

$$\mathrm{sum}\;\mathrm{variance}\;Svarh=\sum_{i=2}^{2N_{g}}{\left(i-Senth\right)}^{2}p_{x+y}(i),$$ (15)

$$\mathrm{sum}\;\mathrm{entropy}\;Senth=-\sum_{i=2}^{2N_{g}}p_{x+y}(i)\log(p_{x+y}(i)),$$ (16)

$$\mathrm{difference}\;\mathrm{variance}\;Dvarh=\sum_{i=0}^{N_{g}-1}i^{2}p_{x-y}(i),$$ (17)

$$\mathrm{difference}\;\mathrm{entropy}\;Denth=-\sum_{i=0}^{N_{g}-1}p_{x-y}(i)\log(p_{x-y}(i)),$$ (18)

$$\mathrm{normalized}\;\mathrm{inverse}\;\mathrm{difference}\;Indnc=\sum_{i}\sum_{j}\frac{p(i,j)}{1+|i-j|/N_{g}^{2}},$$ (19)

normalized inverse difference moment

$$Idmnc=\sum_{i}\sum_{j}\frac{p(i,j)}{1+{\left(i-j\right)}^{2}/N_{g}^{2}},$$ (20)

information measures of correlation

$$Inf1h=\frac{HXY-HXY1}{\max\{HX,HY\}},$$ (21)

$$Inf2h={\left(1-\exp[-2(HXY2-HXY)]\right)}^{\frac{1}{2}},$$ (22)

where $HXY=-\sum_{i}\sum_{j}p(i,j)\log(p(i,j))$, *HX* and *HY* is the entropy of *p*_*x*_ and *p*_*y*_. $HXY1=-\sum_{i}\sum_{j}p(i,j)\log(p_{x}(i)p_{y}(j))$, $HXY2=-\sum_{i}\sum_{j}p_{x}(i)p_{y}(j)\log(p_{x}(i)p_{y}(j))$.

**Fig 1:**
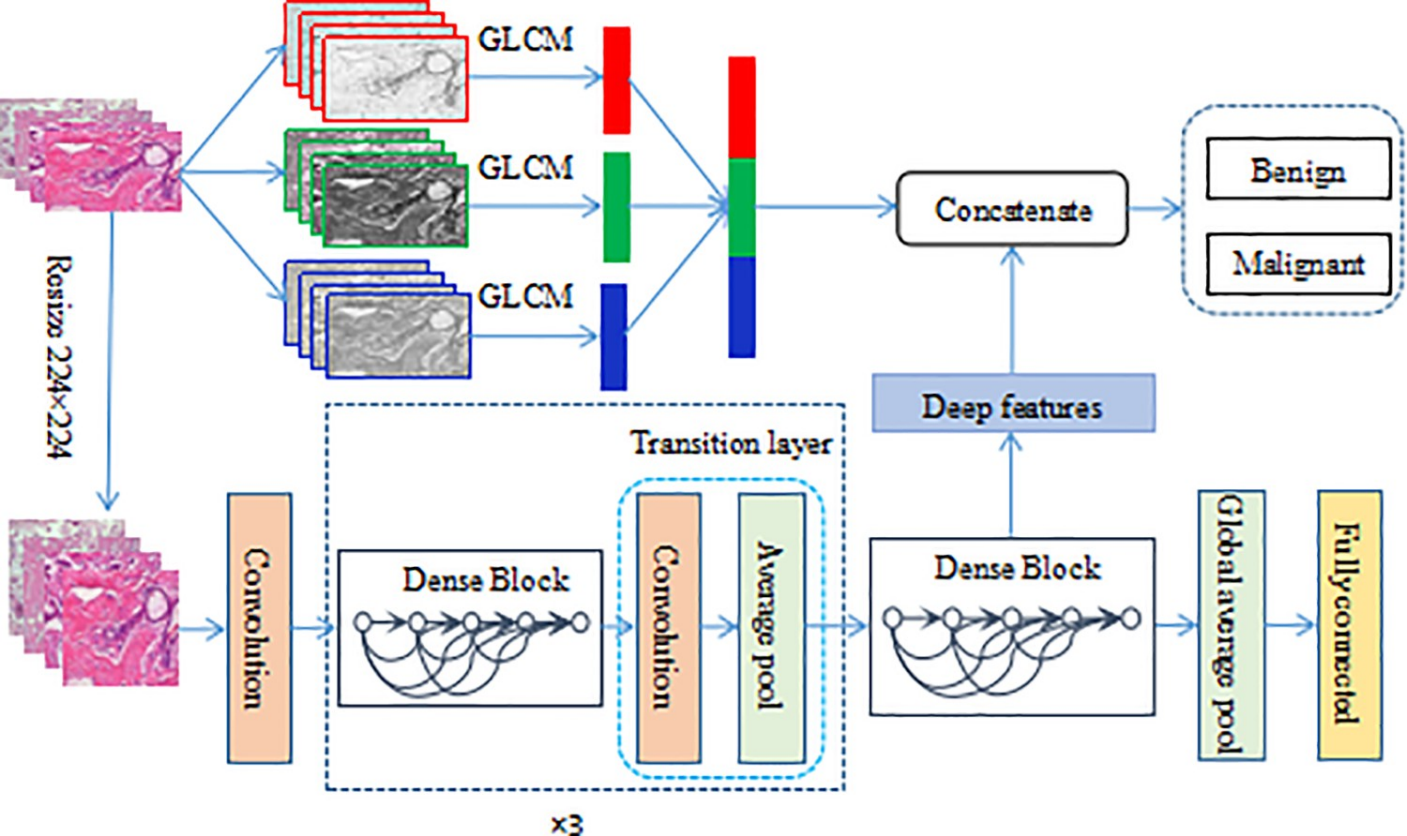
The proposed framework for histopathological images classification.

In this paper, a method of breast cancer histopathological images recognition based on deep semantic features and three-channel GLCM features is presented. The framework is shown in Fig 1. On the one hand, the original images are separated into R, G, and B channels, and the GLCM features of the three channels are extracted respectively. On the other hand, the original images are resized to 224×224, and then input to the pre-trained DensNet201 to extract the deep semantic features. Here, the output of the 1×1 convolutional layer in the 4th, 6th, 14th, 19th, 22nd, and 23rd blocks in the last dense block are extracted as the deep features. Concatenate the obtained three-channel GLCM features and deep semantic features, SVM is used to classify benign and malignant breast cancer.

## Experiments

### Dataset

**Fig 2:**
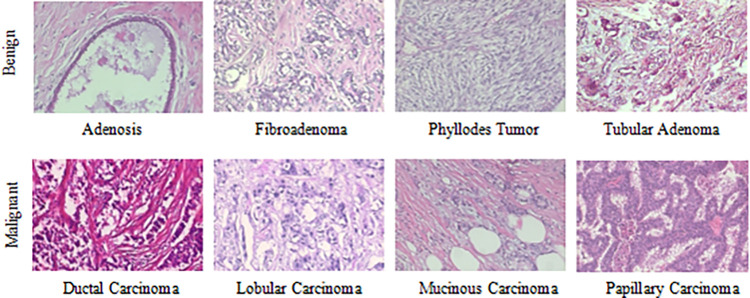
Representative examples with magnification of 100× of BreaKHis dataset.

The BreaKHis dataset [3] contains biopsy images of benign and malignant breast tumors, which were collected through clinical studies from January 2014 to December 2014. During the period, all patients with clinical symptoms of breast cancer were invited to the Brazilian P&D laboratory to participate in the study. Samples were collected by surgical open biopsy (SOB) and stained with hematoxylin and eosin. These images can be used for histological studies and marked by pathologists in the P&D laboratory. The BreaKHis dataset consists of 7909 breast tumor tissue microscopic images of 82 patients, divided into benign and malignant tumors, including 2480 benign (24 patients) and 5429 malignant (58 patients). Each type is further divided into four subclasses. The type benign consist of adenosis (A), fibroadenoma (F), phyllodes tumor (PT) and tubular adenoma (TA) and the type malignant consist of ductal carcinoma (DC), lobular carcinoma (LC), mucinous carcinoma (MC) and papillary carcinoma (PC). The images are obtained in a three-channel RGB (red-green-blue) true color space with magnifications of 40×, 100×, 200×, 400×, and the size of each image is 700×460. Table 1 summarizes the image distribution. And Fig 2 shows the representative examples with magnification of 100× of BreaKHis dataset.

**Table 1:** Image distribution by magnifications and classes.

Magnification	Benign	Malignant	Total
40×	625	1370	1995
100×	644	1437	2081
200×	623	1390	2013
400×	588	1232	1820
Total	2480	5429	7909
Patients	24	58	82

### Implementation details

All of the experiments were conducted on a platform with an Intel Core i7-5820K CPU and 16G random access memory. The BreaKHis dataset has been randomly divided into a training set (70%, 56 patients) and a test set (30%, 26 patients). We guarantee that patients used to build the training set are not used for the test set. Similar to the protocol proposed by Spanhol et al. [3], the dataset was randomly arranged into five folds. The results presented in this work are the average of five trials.

As for MIB classification, we hope to realize the recognition of breast cancer histopathological images without considering the magnifications. The training set for MIB classification is composed of training sets of 40×, 100×, 200×, and 400×, the same for the test set.

**Fig 3:**
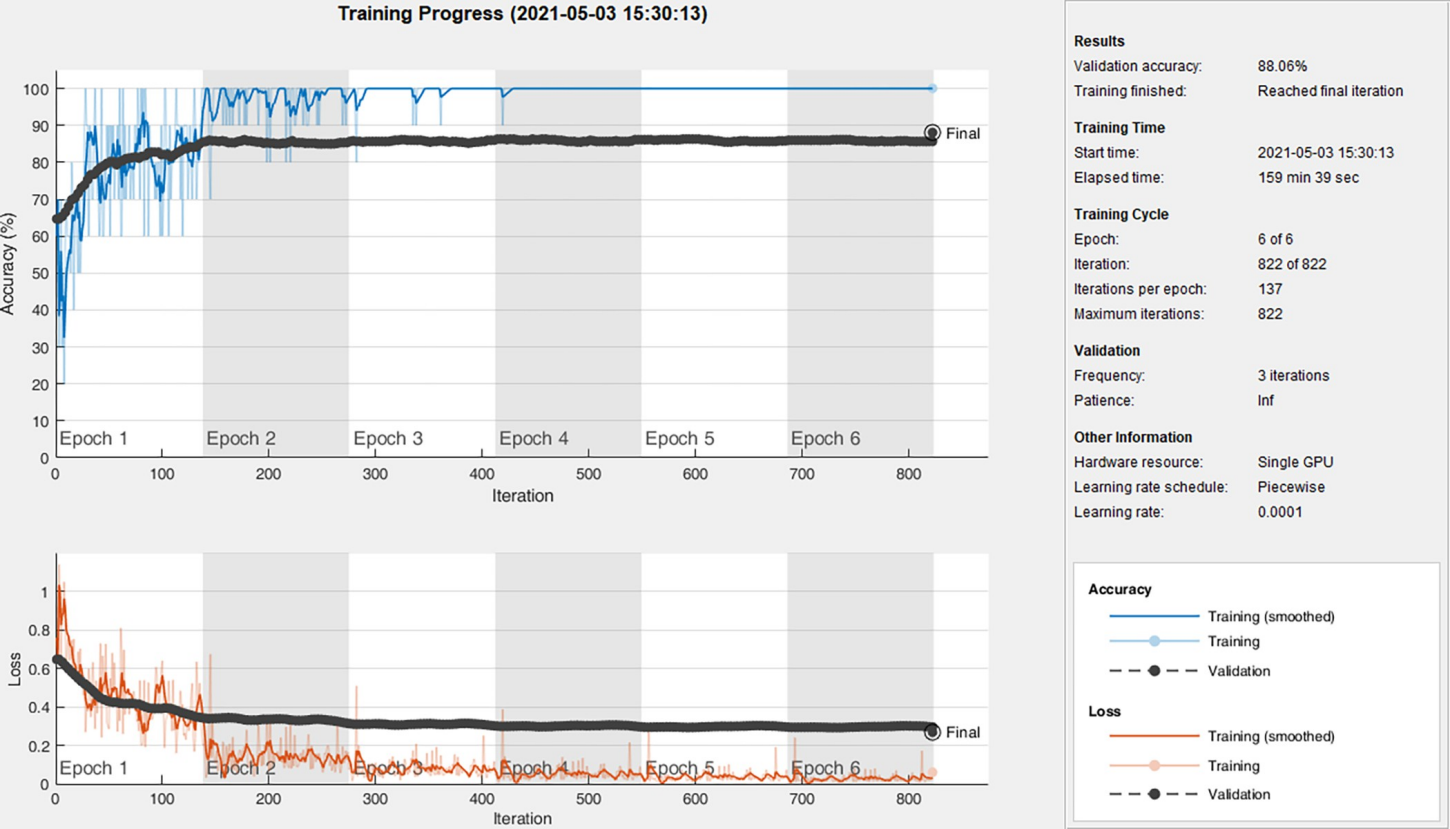
Accuracy and loss curves of DenseNet201 at 40× in fold1.

All the images we used for GLCM features were without any preprocessing. Since different network structures require different sizes of input, to compare with different baseline models, here we resized the images to 224×224, 227×227, 299×299. Among them, the input size of VGG16, ResNet50, GoogLeNet, and DenseNet201 is 224×224, the input size of AlexNet, SqueezeNet is 227×227, and the input size of Inception-ResNet-V2 is 299×299. The baseline models are all well pre-trained on ImageNet and Nvidia GeForce GTX 1080Ti GPU was used for model training. The stochastic gradient descent (SGD) method was used to fine-tune the weights of the entire network for the seven models, the momentum factor is 0.9. The initial learning rate was set as 0.0001 to avoid distorting the initial pre-trained weights as they have been already well tuned. We trained our model for 6 epochs with the minimum batch size of 10 images. The cross-entropy was adopted as the loss function. Taking 40× in fold1 as an example, the accuracy and loss curves of DenseNet201 are given in Fig 3.

The images used for deep feature extraction were also resized to 224×224 in order to reduce the calculation while making a fair comparison with the baseline models. For the SVM, we chose the RBF kernel. The best penalty factor *c* = 2 and kernel function parameter *g* = 1 were obtained by cross validation.

### Evaluation metrics

We report the recognition accuracy at both the image-level and the patient-level. For the image-level, let *N*_rec_I_ be the number of images correctly classified, *N* represents all the test samples, then the recognition accuracy of the image-level can be defined as

$$Image\_accuracy=\frac{N_{rec\_I}}{N}.$$ (23)

For the patient-level, we followed the definition of [3]. Let *N*_*P*_ be the images of patient *P*, *S* is the total number of patients, and *N*_*rec*_*P*_ images of patient *P* were correctly classified, then the patient score can be defined as

$$Patient\;score=\frac{N_{rec\_P}}{N_{P}},$$ (24)

and define the recognition accuracy of the patient-level as

$$Patient\_accuracy=\frac{\sum Patient\;score}{S}.$$ (25)

To further assess the performance of the proposed framework, sensitivity (Se), precision (Pr) and F1_score metrics were used and the formulations of the metrics are described as

$$Se=\frac{TP}{TP+FN},$$ (26)

$$Pr=\frac{TP}{TP+FP},$$ (27)

$$F1\_score=\frac{2\times TP}{2\times TP+FP+FN},$$ (28)

where true positive (TP) represents the number of malignant samples classified as malignant, true negative (TN) represents the number of benign samples classified as benign. Also, false positive (FP) represents the number of benign samples incorrectly classified as malignant while false negative (FN) represents the number of malignant samples misclassified as benign.

## Results

In this part, we separately discussed the results of MSB classification and MIB classification for breast cancer histopathological images recognition.

### Magnification specific binary classification

Firstly, through comparative analysis, we find that the features of 1×1 convolutional layer in the 4th, 6th, 14th, 19th, 22nd and 23rd blocks in the last dense block are more discriminative. For the sake of description, we use the following naming method: Dense Block4_block4_1 means to extract the output of the 1×1 convolutional layer in the 4th block of the 4th dense block as features. In this paper, we extracted the features of the following convolutional layers: Dense Block4_block4_1, Dense Block4_block6_1, Dense Block4_block14_1, Dense Block4_block19_1, Dense Block4_block22_1, Dense Block4_block23_1, which are short as block4, block6, block14, block19, block22, block23 in the following description. Table 2 shows the comparison of classification performance of convolutional layer features with pooling layer features and fully connected layer features.

**Table 2:** Classification performance of different deep layer features.

Features	Magnification	Image_accuracy((%)	Patient_accuracy(%)	Sensitivity(%)	Precision(%)	F1_score(%)
Average pool_1	40×	89.17±4.48	88.20±5.10	87.48±5.15	97.56±2.24	92.21±3.59
	100×	90.68±1.61	91.08±2.66	89.32±1.66	98.01±1.88	93.46±1.43
	200×	90.71±2.00	89.94±2.27	91.42±0.81	96.05±2.86	93.66±1.44
	400×	86.68±2.75	86.17±3.11	87.58±1.15	93.83±3.69	90.57±2.08
Average pool_2	40×	91.91±2.93	92.13±2.89	90.45±3.81	98.48±0.95	94.27±2.33
	100×	92.46±2.75	93.29±2.72	91.68±3.31	98.00±1.45	94.72±2.19
	200×	92.01±2.59	91.57±2.54	92.29±2.81	96.91±2.91	94.51±1.94
	400×	88.89±1.49	88.46±2.31	88.64±1.70	95.97±3.25	92.12±1.07
Average pool_3	40×	92.11±2.67	91.91±2.96	91.57±2.17	97.75±1.56	94.55±1.82
	100×	92.56±3.09	92.93±3.18	92.30±3.69	97.46±1.20	94.79±2.46
	200×	92.20±1.79	91.90±2.02	91.97±3.04	97.49±1.69	94.61±1.50
	400×	89.16±2.17	88.37±2.30	89.13±3.97	95.78±2.43	92.27±1.92
Global average pool	40×	90.82±2.28	90.24±2.89	89.89±2.73	97.59±1.17	93.57±1.81
	100×	90.02±3.06	90.00±3.08	89.60±3.57	96.72±1.39	93.00±2.46
	200×	89.21±3.40	88.44±3.83	88.78±4.23	96.49±1.50	92.44±2.74
	400×	86.99±2.42	86.41±2.61	87.36±3.60	94.40±1.57	90.71±2.13
FC	40×	89.08±1.76	88.39±2.09	88.10±3.02	96.95±1.04	92.29±1.70
	100×	88.97±2.65	89.35±2.61	88.41±3.40	96.43±1.18	92.23±2.30
	200×	89.10±3.16	88.42±3.64	89.14±3.67	95.96±1.52	92.40±2.55
	400×	85.66±2.27	84.94±2.20	86.17±3.28	93.68±1.71	89.74±2.05
block4	40×	90.05±2.41	90.12±2.19	90.41±2.33	95.97±1.67	93.10±1.90
	100×	91.99±2.60	92.31±3.10	92.64±3.48	96.42±1.81	94.47±2.10
	200×	91.26±1.41	91.32±1.33	92.47±2.36	95.84±3.20	94.07±1.10
	400×	89.98±1.42	89.59±1.33	90.60±2.45	95.61±3.57	92.97±1.12
block6	40×	91.57±1.22	90.40±3.10	91.88±1.56	96.67±1.43	94.2±0.98
	100×	91.31±2.10	91.71±2.76	92.39±2.50	95.78±1.83	94.04±1.72
	200×	91.26±2.60	91.36±2.68	92.67±3.17	95.62±3.71	94.05±1.96
	400×	89.62±2.04	89.18±2.05	89.66±2.32	95.85±2.45	92.63±1.72
block14	40×	91.94±1.71	91.92±1.47	90.96±2.43	98.12±0.56	94.39±1.27
	100×	91.96±2.44	92.35±3.00	91.97±3.60	97.03±1.49	94.40±2.05
	200×	93.32±1.88	93.19±2.41	93.73±3.16	97.27±1.79	95.43±1.51
	400×	90.29±2.51	89.89±2.70	90.79±3.00	95.66±2.62	93.13±2.09
block19	40×	90.94±3.29	90.57±3.78	90.58±3.50	97.10±1.40	93.71±2.34
	100×	92.20±1.96	92.61±2.64	92.35±3.29	97.05±1.78	94.60±1.70
	200×	93.24±1.83	92.95±1.53	94.34±2.83	96.64±3.14	95.42±1.38
	400×	91.03±1.71	90.76±1.83	91.51±2.11	96.05±2.65	93.70±1.44
block22	40×	92.90±2.02	92.80±2.51	92.82±1.79	97.57±1.36	95.13±1.40
	100×	92.25±2.53	92.82±2.80	92.04±2.97	97.34±1.52	94.60±2.06
	200×	92.70±2.38	92.60±2.66	92.68±3.66	97.44±1.58	94.96±1.91
	400×	90.35±1.88	89.82±2.12	90.98±2.46	95.61±2.63	93.2±1.60
block23	40×	92.49±2.28	92.47±2.32	92.13±2.61	97.67±0.90	94.81±1.70
	100×	92.45±2.04	92.84±2.56	92.50±2.63	97.19±1.40	94.77±1.70
	200×	93.40±1.67	93.33±1.58	93.92±3.10	97.23±2.34	95.49±1.34
	400×	90.29±2.46	89.61±2.85	90.30±2.47	96.17±3.04	93.11±2.01

Comparing the classification performance of different layer features under four magnifications, it can be found that the performance of fully connected layer features is worse than that of pooling layer features and deep convolutional layer features, and the recognition accuracy of four magnifications are all less than 90%. The recognition accuracy of the features of the pooling layers at 400× is significantly lower than that of the convolutional layers. This is because the images at 400× contain more accurate lesion information, which is often local information. The pooling operation loses part of the spatial information of the images, which makes cancer detection more difficult. Compared with the fully connected layer and pooling layer features, the convolutional layer features retain the spatial and structural information of the images. It can be seen from Table 2 that the convolutional layer features perform well for the images under four magnifications. The recognition accuracy are all higher than 90% except for block4 and block6 at 400×, and the performance of block14, block19 and block23 is better. In addition, it is verified by experiments that with the deepening of the network, the features become more and more abstract, the classification performance showed a downward trend. So the convolutional layer features deeper than block23 are no longer considered here.

**Fig 4:**
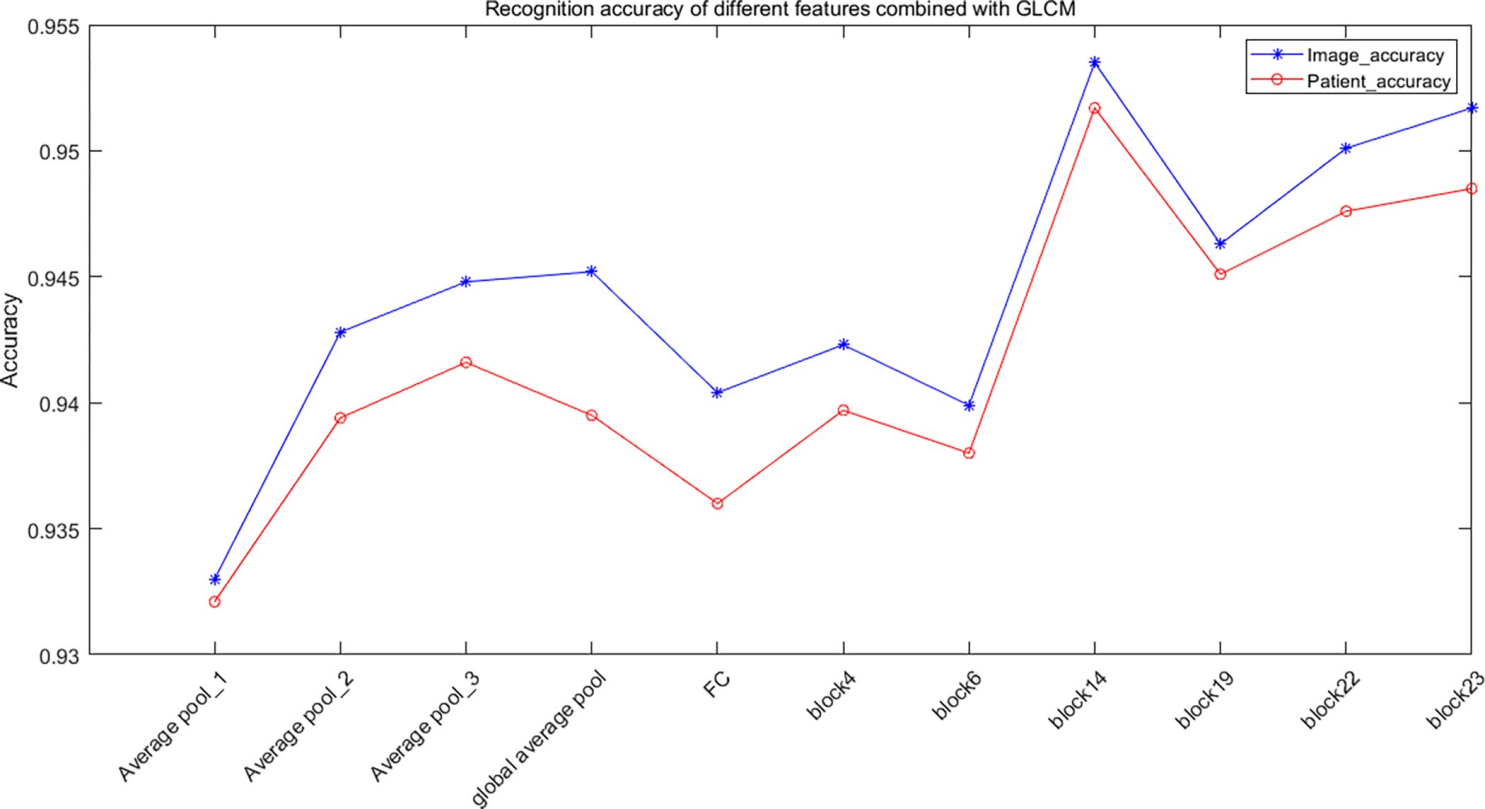
Recognition accuracy of different features combined with GLCM.

Table 3 shows the classification performance of fused features of deep semantic features and GLCM features for MSB classification. Comparing Tables 2 and 3, it can be found that the classification performance based on the combination of deep semantic features and GLCM is significantly better than the classification performance of deep semantic features. The highest recognition accuracy at the image-level is 96.75%, 95.21%, 96.57%, and 93.15% for 40×, 100×, 200×, 400×, respectively. And the highest recognition accuracy at the patient-level is 96.33%, 95.26%, 96.09%, and 92.99%, respectively. Compared with pooling layer and fully connected layer features, the fused features of convolutional layer features and GLCM achieve higher accuracy, as shown in Fig 4. Although the recognition accuracy of Average pool_3 is better than that of block4 and block6, its recognition time is about 15–20 times that of convolutional layer features.

**Table 3:** MSB classification performance of fused features.

Features	Magnification	Image_accuracy(%)	Patient_accuracy(%)	Sensitivity(%)	Precision(%)	F1_score(%)
Average pool_1+GLCM	40×	93.26±2.73	93.30±3.12	94.13±2.95	90.98±5.60	96.71±2.28
	100×	92.78±1.90	93.03±3.18	93.64±2.86	90.88±7.78	96.62±2.95
	200×	95.06±1.57	94.38±1.49	95.78±2.23	92.86±5.52	97.59±1.80
	400×	92.08±2.74	92.11±2.97	95.47±2.89	88.63±9.00	95.67±3.66
Average pool_2+GLCM	40×	94.86±3.02	94.11±3.38	96.93±3.17	93.07±5.37	97.45±2.15
	100×	94.61±2.22	94.12±2.50	94.61±2.70	95.81±2.33	98.42±1.08
	200×	94.99±2.00	95.09±2.35	96.53±2.01	94.42±5.48	98.08±1.83
	400×	92.65±2.32	92.42±2.12	95.33±1.91	87.87±6.64	95.41±2.83
Average pool_3+GLCM	40×	95.02±1.97	94.92±2.20	97.01±2.66	94.16±3.96	98.01±1.32
	100×	94.91±1.99	94.60±2.32	97.14±1.83	91.70±3.52	97.09±1.57
	200×	95.62±1.98	95.21±2.08	97.19±2.20	91.96±3.18	97.32±1.15
	400×	92.35±2.26	91.90±3.08	93.03±2.97	90.44±3.21	96.31±1.51
Global average pool+GLCM	40×	95.02±1.87	94.53±2.50	97.22±1.20	92.47±4.43	97.44±1.49
	100×	94.75±2.62	94.05±3.21	96.25±2.61	92.69±3.37	97.39±1.50
	200×	95.38±1.89	95.01±2.23	96.68±2.91	96.90±2.54	98.96±0.86
	400×	92.91±2.41	92.20±2.62	93.64±2.81	93.99±4.09	97.56±1.91
FC+GLCM	40×	94.36±2.02	94.17±2.38	96.70±2.26	91.23±4.48	97.05±1.46
	100×	94.13±1.99	93.45±2.68	96.83±2.68	85.56±5.59	95.35±1.55
	200×	95.55±1.22	95.35±1.41	97.16±1.67	91.84±4.95	97.31±1.65
	400×	92.13±2.54	91.42±2.43	93.65±2.08	88.07±3.84	95.40±2.12
block4+GLCM	40×	94.32±2.76	93.80±2.78	96.11±2.32	96.19±1.95	96.14±2.03
	100×	94.31±2.72	94.27±2.90	96.36±2.59	95.91±1.77	96.13±2.08
	200×	95.52±1.77	95.28±1.81	96.74±1.74	97.29±2.46	96.99±1.28
	400×	92.77±2.50	92.53±2.40	95.00±1.83	95.05±2.75	95.01±1.91
block6+GLCM	40×	94.09±1.81	93.66±1.88	95.93±1.30	96.12±1.88	96.02±1.35
	100×	93.60±2.24	93.71±2.40	95.75±2.22	95.59±1.66	95.67±1.79
	200×	95.36±1.92	95.07±1.76	97.40±1.43	96.44±2.25	96.90±1.39
	400×	92.90±1.98	92.78±2.08	94.23±2.33	95.95±2.17	95.06±1.61
block14+GLCM	40×	96.75±1.96	96.33±2.14	97.86±1.53	97.76±2.03	97.80±1.41
	100×	95.20±2.31	95.26±2.60	95.79±2.11	97.63±1.65	96.69±1.78
	200×	96.29±1.49	96.09±1.79	97.14±1.77	97.86±1.29	97.49±1.12
	400×	93.15±2.30	92.99±2.85	94.43±2.51	96.09±2.42	95.23±1.83
block19+GLCM	40×	94.86±2.31	94.50±2.64	95.93±2.39	97.10±1.38	96.51±1.69
	100×	94.87±2.11	95.07±2.19	96.53±1.49	96.54±2.37	96.53±1.61
	200×	95.79±1.71	95.48±1.47	97.81±1.48	96.64±2.48	97.20±1.19
	400×	93.02±2.35	92.99±2.39	95.07±2.77	95.34±2.51	95.17±1.84
block22+GLCM	40×	96.29±1.82	95.87±2.11	98.52±1.68	96.53±1.43	97.51±1.32
	100×	94.93±2.27	94.90±2.35	96.57±1.49	96.56±2.04	96.56±1.71
	200×	95.73±2.09	95.48±2.16	96.77±2.21	97.48±1.80	97.11±1.54
	400×	93.09±2.31	92.86±2.23	94.19±2.60	96.22±2.06	95.17±1.83
block23+GLCM	40×	95.86±1.73	95.49±1.51	96.90±1.90	97.48±1.38	97.18±1.31
	100×	95.21±2.18	95.23±2.61	95.99±2.30	97.47±1.68	96.71±1.69
	200×	96.57±1.82	96.05±1.94	97.40±2.35	97.97±1.47	97.67±1.35
	400×	93.05±2.05	92.63±2.82	93.98±2.50	96.40±2.41	95.14±1.67

**Fig 5:**
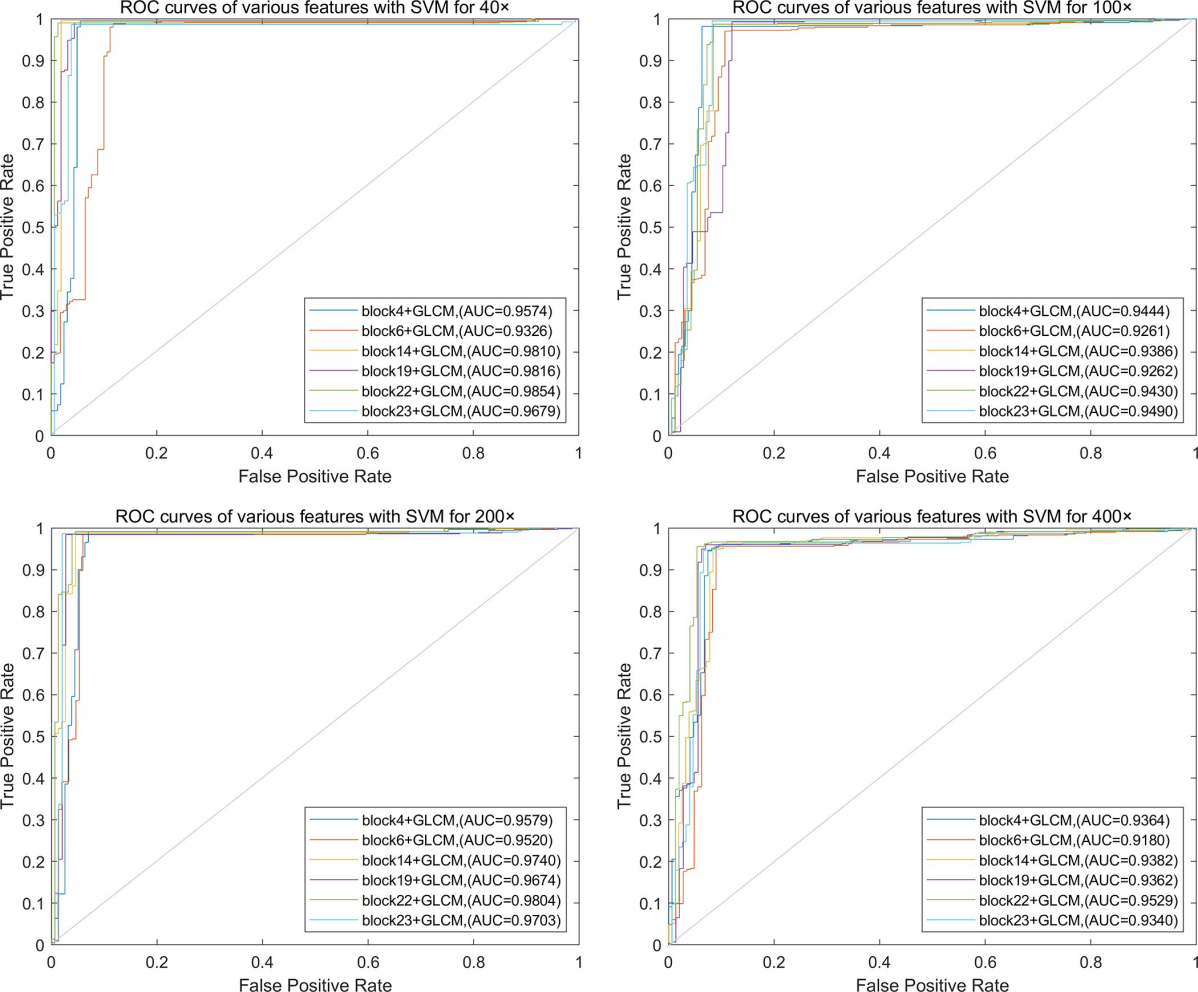
ROC curves of different feature combinations for MSB classification. (a) 40×, (b) 100×, (c) 200×, (d) 400×.

Based on the above conclusion, the fused features of convolutional layer features and GLCM for breast cancer histopathological images recognition is discussed below. The Receiver Operating Characteristic (ROC) curves of classification performance of different feature combinations are shown in Fig 5.

**Fig 6:**
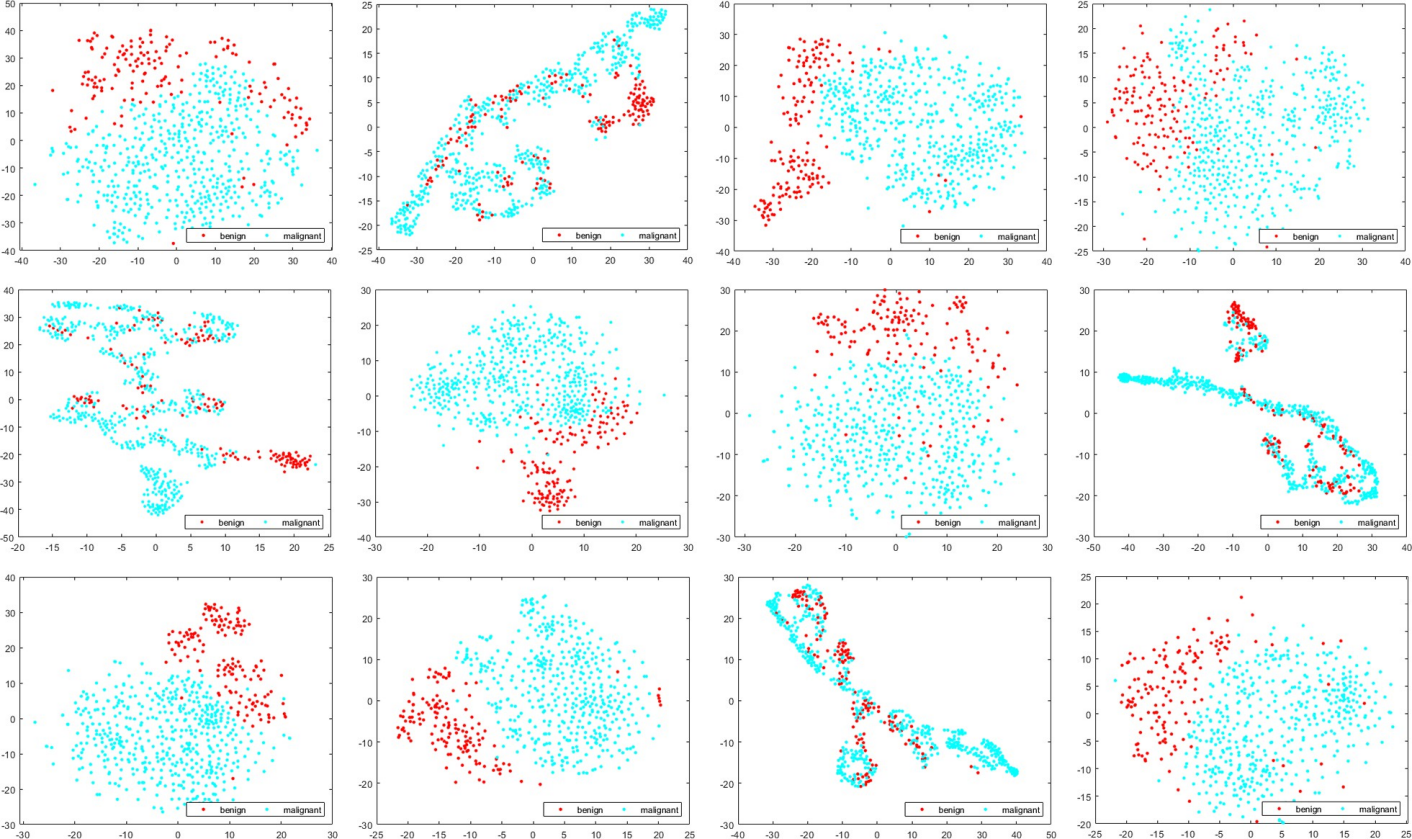
Visualization of different features for MSB classification.

In order to investigate the classification performance of different deep semantic features and GLCM, we use t-distributed stochastic neighbor embedding (t-SNE) to visualize deep semantic features, GLCM features and the fused features, as shown in Fig 6 (Take block14 as an example).

In Fig 6, each row represents different magnifications, 40×: (a)-(c), 100×: (d)-(f), 200×: (g)-(i), 400×: (j)-(l); Each column represents different types of features, the first column represents the first convolutional layer features of block14, the second column represents GLCM features, and the third column represents the fused features of deep semantic features and GLCM features. As shown in Fig 6, the separability of GLCM features is poor, features of different classes are interlaced. The second is deep semantic features. Although there is a clear boundary between the two classes of features, the distribution of features is scattered. The separability of the fused features is the best. The two classes of features are clearly separated and concentrated, with only a small number of samples interlaced. In addition, comparing different magnifications, it can be found that the features of 200× has the best separability, so as to obtain the highest recognition accuracy.

To further illustrate the effectiveness of the proposed method, we compared the performance of seven pre-trained baseline models for breast cancer binary classification, as shown in Table 4.

**Table 4:** MSB classification performance of baseline models.

Methods	Magnification	Image_accuracy(%)	Patient_accuracy(%)	Sensitivity(%)	Precision(%)	F1_score(%)
AlexNet	40×	86.38±3.46	87.21±3.85	87.48±6.60	93.85±2.22	90.41±3.16
	100×	88.17±3.52	88.66±3.35	87.23±4.25	96.55±1.93	91.62±2.82
	200×	89.48±3.38	88.67±3.45	90.79±5.80	94.93±1.28	92.72±2.79
	400×	89.13±3.18	88.50±3.11	91.10±5.21	93.87±2.99	92.37±2.65
VGG16	40×	90.26±2.75	90.50±2.96	91.35±4.47	95.50±2.48	93.30±2.02
	100×	90.06±4.83	89.40±4.54	91.25±4.96	95.11±2.75	93.11±3.64
	200×	93.87±2.00	93.15±2.88	93.88±2.51	97.84±1.08	95.80±1.50
	400×	90.82±2.62	89.83±1.99	93.74±2.31	93.75±3.43	93.71±1.92
ResNet50	40×	90.24±4.27	90.39±4.54	90.64±5.32	95.87±2.08	93.14±3.52
	100×	92.09±3.28	92.17±3.21	94.10±3.16	95.13±2.63	94.60±2.52
	200×	91.04±2.42	89.57±2.28	93.01±3.09	94.94±1.33	93.94±1.80
	400×	88.75±2.56	87.96±2.55	91.23±5.79	93.42±3.35	92.15±2.28
GoogLeNet	40×	91.61±3.16	91.29±3.30	91.66±3.83	96.87±2.14	94.16±2.47
	100×	91.73±3.42	91.97±3.76	91.82±5.03	96.82±0.90	94.20±2.79
	200×	92.00±2.31	90.72±2.47	92.90±3.96	96.32±2.33	94.52±1.88
	400×	90.39±2.28	89.61±2.45	90.74±3.32	96.04±4.07	93.22±1.82
SqueezeNet	40×	86.99±1.53	85.69±3.06	87.91±5.38	94.54±3.19	90.94±1.46
	100×	91.35±3.99	91.04±4.22	93.73±3.74	94.51±2.84	94.10±2.98
	200×	93.09±1.22	92.57±2.37	94.59±1.98	96.20±2.40	95.36±0.92
	400×	88.89±4.20	87.93±4.30	89.76±7.25	94.70±1.72	92.01±3.68
DenseNet201	40×	88.23±2.84	88.62±2.95	87.45±3.70	96.36±1.89	91.66±2.46
	100×	90.38±3.83	90.84±3.70	90.49±5.99	96.40±2.29	93.24±3.16
	200×	91.03±2.46	90.14±2.72	90.69±3.92	97.14±1.63	93.76±2.01
	400×	89.23±2.43	88.78±2.44	89.55±4.38	95.48±2.22	92.34±2.09
Inception-ResNet-V2	40×	88.14±2.44	88.29±4.15	93.05±3.09	91.43±4.23	92.14±1.67
	100×	91.27±0.90	91.16±0.76	95.21±2.55	93.33±3.18	94.20±0.76
	200×	90.66±0.66	89.74±1.38	95.11±3.46	92.86±3.34	93.88±0.34
	400×	87.93±1.90	87.25±2.12	91.80±2.43	91.91±4.38	91.76±1.34

It can be seen from Table 4 that under the same training conditions, as a whole, GoogLeNet and VGG16 performs well under the four magnifications. VGG16 obtained the highest image-level recognition accuracy of 93.87% and the highest patient-level recognition accuracy of 93.15% at 200×. Compared with the performance of DenseNet201, ResNet50 performs better at 40×, 100× and 200×, SqueezeNet performs better at 100× and 200×, and Inception-ResNet-V2 performs better at 100×. AlexNet performs worse than other baseline models, and the performance of DenseNet201 is at an intermediate level. All models perform best at 200×, followed by 100×, indicating that the images with these two magnifications not only contain enough global information, but also contain rich local information, which are more suitable for automatic breast cancer recognition.

In this paper, what we want to achieve is the fusion of deep semantic features and GLCM features. At first, we need to ensure the depth of the network to extract effective deep semantic features. And then the amount of network parameters and the dimension of the extracted features are considered. DenseNet201 has a deeper structure and fewer parameters than AlexNet, VGG16, ResNet50 and Inception-ResNet-V2. For example, in VGG16, with the increase of layers, the dimension of the extracted convolution layer features continues to increase according to the characteristics of network structure, which far exceeds the dimension of GLCM, so that the role of GLCM in the fused features is ignored, resulting in much worse recognition results for fused features than a single VGG16. DenseNet201 not only makes full use of information from different layers, but also limits the dimension of features through 1×1 convolution operations. Deep semantic features and GLCM features give full play to their respective advantages, so as to achieve better recognition results. Therefore, we chose DenseNet201 as the feature extractor.

**Fig 7:**
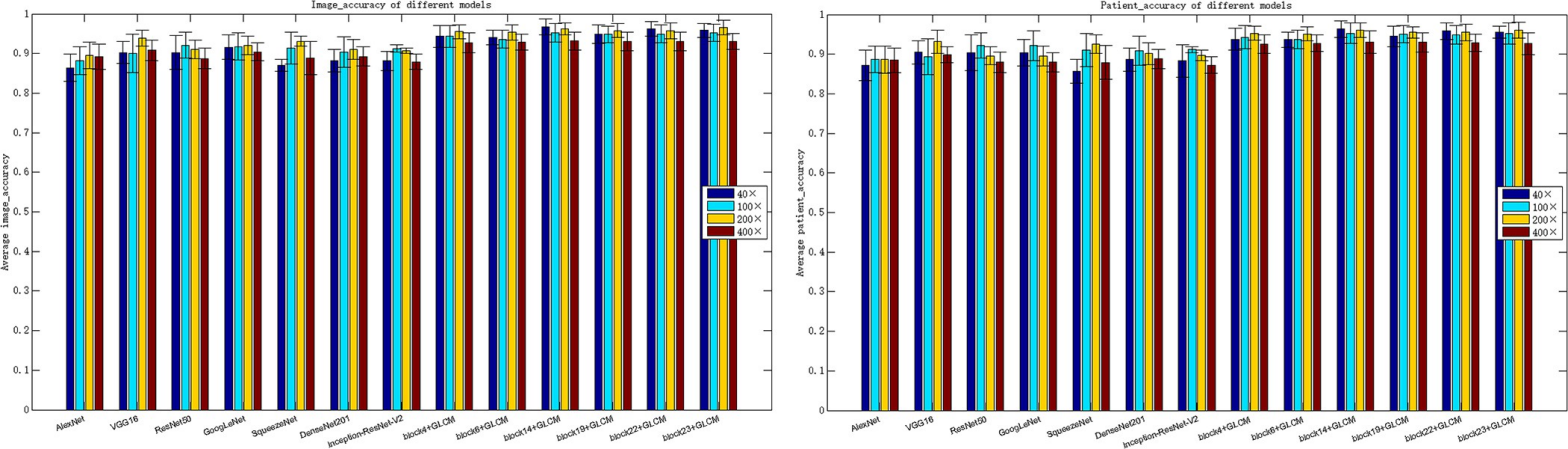
Comparison of the baseline models with the method proposed in this paper. (a) Image-level recognition accuracy, (b) patient-level recognition accuracy.

The recognition accuracy of all baseline models is lower than the method proposed in this paper. The comparison results are shown in Fig 7.

### Magnification independent binary classification

In this section, we discuss the results of MIB classification. Table 5 shows the results of MIB classification.

**Table 5:** MIB classification performance of fused features.

Features	Image_accuracy(%)	Patient_accuracy(%)	Sensitivity(%)	Precision(%)	F1_score(%)
block4+GLCM	94.41±2.20	94.06±2.13	96.77±1.55	95.71±2.03	96.23±1.63
block6+GLCM	93.99±2.07	93.56±2.15	95.87±2.20	95.98±1.55	95.92±1.62
block14+GLCM	95.56±2.14	95.54±2.40	96.62±1.71	97.35±2.10	96.98±1.59
block19+GLCM	94.65±2.02	94.60±2.13	96.80±2.00	95.99±1.63	96.38±1.54
block22+GLCM	94.91±2.69	94.63±2.93	96.11±2.66	96.93±1.65	96.51±2.03
block23+GLCM	95.23±2.10	95.10±2.44	96.06±1.95	97.41±1.48	96.73±1.61

**Fig 8:**
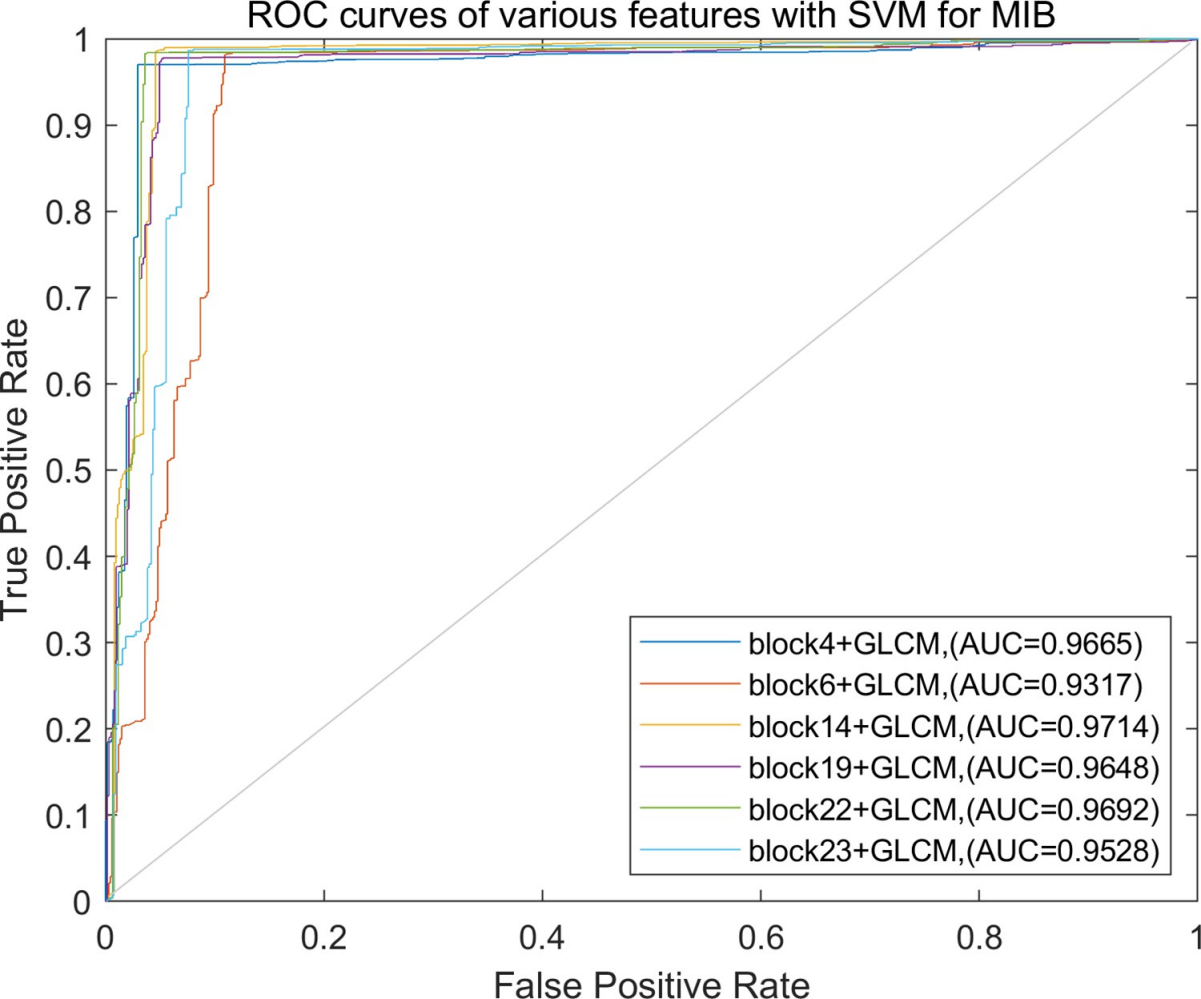
ROC curves of different feature combinations for MIB classification.

Regardless of the magnifications, the recognition accuracy of the method proposed in this paper is still acceptable, especially for block14+GLCM, the image-level recognition accuracy is 95.56%, and the patient-level recognition accuracy is 95.54%, followed by block23+GLCM, the image-level and the patient-level recognition accuracy are 95.23% and 95.10%, respectively. The ROC curves of the recognition performance of different fused features are shown in Fig 8.

Table 6 shows that the performance of the baseline models for MIB classification. It can be seen from Table 6 that GoogLeNet performs best among the models, followed by ResNet50 and DenseNet201. The performance of the method proposed in this paper is significantly better than the baseline models. Comparing Tables 4 and 6, it can be found that the baseline models are not sensitive to the magnifications. The recognition accuracy does not fluctuate much whether the magnification is considered or not.

**Table 6:** MIB classification performance of baseline models.

Methods	Image_accuracy (%)	Patient_accuracy(%)	Sensitivity(%)	Precision(%)	F1_score(%)
AlexNet	89.00±3.97	88.40±3.31	88.44±6.81	96.43±0.97	92.13±3.42
VGG16	89.38±3.01	88.26±3.19	90.25±4.07	95.21±2.84	92.61±2.40
ResNet50	90.84±3.25	90.22±2.93	91.20±4.40	96.17±0.99	93.58±2.62
GoogLeNet	91.98±2.38	91.46±2.32	92.56±3.78	96.58±2.75	94.46±1.85
SqueezeNet	89.37±3.56	88.47±3.63	88.80±6.02	96.61±1.64	92.44±2.96
DenseNet201	90.79±4.16	90.34±3.63	89.91±5.91	97.38±0.94	93.42±3.38
Inception-ResNet-V2	88.95±2.34	88.18±2.18	91.56±2.12	93.46±1.97	92.49±1.76

### Comparison of experimental results

Table 7 is a comparison between the method in this paper and the state-of-the-art methods. Works [45, 46] divided the dataset according to the protocol of [3], works [31, 47] divided the dataset according to the patients, the author in [48] divided the dataset according to the images in the image-level classification, the authors in [39, 40] divided the dataset according to the images, and works [49–51] did not mention whether to divide the dataset according to the patients or the images.

**Table 7:** Comparison of results between proposed method and the state-of-the-art methods.

Methods	Training/Test	Magnification	Image_accuracy(%)	Patient_accuracy(%)
Gour et al. [45]	70/30 (protocol)	40×	87.40±3.00	87.47±3.22
		100×	87.26±3.54	88.15±2.97
		200×	91.15±2.30	92.52±2.84
		400×	86.27±2.18	87.78±2.46
Alkassar et al. [46]	70/30 (protocol)	40×	99	
		100×	98.5	
		200×	98.5	
		400×	98	
Li et al. [31]	50/20/30	40×	89.5±2.0	89.1±3.6
		100×	87.5±2.9	85.0±5.1
		200×	90.0±5.3	87.0±6.0
		400×	84.0±2.9	84.5±3.6
Sharma et al. [47]	80/20	40×	89.31	
		100×	85.75	
		200×	83.95	
		400×	84.33	
Celik et al. [48]	80/20	Magnification independent	99.11	89.88
Yari et al. [39]	6011/1142/406	40×	100	
		100×	100	
		200×	98.08	
		400×	98.99	
		Magnification independent	99.26	
Liu et al. [40]	Random 5 folds	40×	99.33	
		100×	99.04	
		200×	98.84	
		400×	98.53	
		Magnification independent	99.24	
Budak et al. [49]	Random 5 folds	40×	95.69±1.78	
		100×	93.61±2.28	
		200×	96.32±0.51	
		400×	94.29±1.86	
Mewada et al. [50]	Random 70/30	40×	97.58	
		100×	97.44	
		200×	97.28	
		400×	97.02	
Nahid et al. [51]	Didn’t mention	40×	90	
		100×	85	
		200×	90	
		400×	91	
Our method	70/30	40×	96.75±1.96	96.33±2.14
		100×	95.21±2.18	95.26±2.60
		200×	96.57±1.82	96.09±1.79
		400×	93.15±2.30	92.99±2.85
		Magnification independent	95.56±2.14	95.54±2.40

It can be seen from Table 7 that the classification performance of the methods which dividing the dataset according to the images is significantly better than our method. The recognition accuracy of our methods is significantly higher than other methods that dividing the dataset according to the patients except for [46, 50], but there is still room for improvement.

## Discussion

From the experimental results, we can see that the method proposed in this paper is very effective in classifying the breast cancer for both the MSB classification and MIB classification. Compared with the pooling layer features and the fully connected layer features, the convolutional layer features retain more spatial and structural information of the images, and show better separability, which is beneficial to the recognition of breast cancer. In addition, we discussed the classification problem of breast cancer which does not depend on magnifications. It does not need to consider the magnification of the images in this method, and avoids the trouble of training multiple models with different magnifications, which ensures the high accuracy of recognition while improving the efficiency of the model training. It is meaningful in practical application. Given an unlabeled image, what we need to do is to identify whether it is benign or malignant, while without considering its magnification.

A commonly used method in existing works is model training based on image patches. Firstly, the original images need to be divided into small image patches, the labels of the image patches are predicted one by one, and then the labels of the image patches are integrated to predict the image label. There is a problem with this method. For a malignant image, the malignant tissues are not full of the whole image, it often contains some benign tissues. Using the label of the original image as the label of the image patches cannot guarantee the consistency of the label and often reduces the recognition accuracy. It is very time-consuming for getting image patches, and the classification performance of the model also depends on the size of the image patches. There are also some researchers who use data augmentation to increase the diversity of the samples when training the model, but for pre-trained models, we only need to adjusted some of the parameters. Although we did not consider data augmentation, there is no over-fitting problem in our method.

In view of this, we used the original images for GLCM feature extraction, and used the resized images to fine-tune the pre-trained models. GLCM provides the texture features of the images, DenseNet201 makes full use of the features of different layers, and the features of the deep convolutional layer retain more spatial information of the images. These features are complementary to each other and achieve better recognition performance.

## Conclusion

In this paper, a breast cancer histopathological images classification method based on the fusion of DenseNet201 deep semantic features and three-channel GLCM features is proposed. Unlike other methods that only consider the features of the pooling layers and the fully connected layers of the CNN models, we explored the discriminative ability of different deep convolutional layer features, and fused the extracted deep semantic features with three-channel GLCM features for breast cancer histopathological images MSB and MIB classification. For the four magnifications, the highest recognition accuracy of the image-level is 96.75%, 95.21%, 96.57%, 93.15%, respectively, and the highest recognition accuracy of the patient-level is 96.33%, 95.26%, 96.09%, 92.99%, respectively. The accuracy of the image-level and the patient-level for MIB classification is 95.56% and 95.54%, respectively. Experimental results show that the method proposed in this paper is robust to the two classification problems. The comparison results with seven baseline models indicate that the performance of the method proposed in this paper is better.

In the future work, we will continue to study the multi-class recognition of breast cancer histopathological images and realize the sub-class recognition of breast cancer, which can provide more accurate theoretical basis for pathologists, and to further reduce their workload.
